# Cholesterol Metabolism: A Double-Edged Sword in Hepatocellular Carcinoma

**DOI:** 10.3389/fcell.2021.762828

**Published:** 2021-11-10

**Authors:** Fangli Zhou, Xiaoli Sun

**Affiliations:** ^1^ Department of Endocrinology and Metabolism, West China Hospital, Sichuan University, Chengdu, China; ^2^ Department of Pharmacology, Mays Cancer Center, Transplant Center, University of Texas Health Science Center at San Antonio, San Antonio, TX, United States

**Keywords:** hepatocellular carcinoma, nonalcoholic fatty liver disease, nonalcoholic steatohepatitis, cholesterol metabolism, tumor microenvironment, lipotoxicity, fibrosis, inflammation

## Abstract

Hepatocellular carcinoma (HCC) represents a leading cause of cancer-related deaths globally. The rising incidence of metabolic syndrome and its hepatic manifestation, nonalcoholic fatty liver disease (NAFLD), have emerged as the fastest-growing cause of HCC in recent years. Cholesterol, a major lipid component of the cell membrane and lipoprotein particles, is primarily produced and metabolized by the liver. Numerous studies have revealed an increased cholesterol biosynthesis and uptake, reduced cholesterol exportation and excretion in HCC, which all contribute to lipotoxicity, inflammation, and fibrosis, known HCC risk factors. In contrast, some clinical studies have shown that higher cholesterol is associated with a reduced risk of HCC. These contradictory observations imply that the relationship between cholesterol and HCC is far more complex than initially anticipated. Understanding the role of cholesterol and deciphering the underlying molecular events in HCC development is highly relevant to developing new therapies. Here, we discuss the current understanding of cholesterol metabolism in the pathogenesis of NAFLD-associated HCC, and the underlying mechanisms, including the roles of cholesterol in the disruption of normal function of specific cell types and signaling transduction. We also review the clinical progression in evaluating the association of cholesterol with HCC. The therapeutic effects of lowering cholesterol will also be summarized. We also interpret reasons for the contradictory observations from different preclinical and human studies of the roles of cholesterol in HCC, aiming to provide a critical assessment of the potential of cholesterol as a therapeutic target.

## Introduction

Primary liver cancer ranks the fifth and ninth most commonly diagnosed cancer in men and women, respectively, and the third most fatal cancer globally (Ferlay et al., 2015; Sung et al., 2021). Hepatocellular carcinoma (HCC) is the most common type of primary liver cancer. Although Hepatitis B virus (HBV) and hepatitis C virus (HCV) are still the top risk factors for HCC, a large percentage of HCC arises due to nonalcoholic fatty liver disease (NAFLD) (El-Serag and Kanwal, 2014; Chaitanya Thandra et al., 2020). The prevalence of NAFLD is increasing because of the global epidemics of metabolic syndrome, a cluster of conditions including hypercholesterolemia, hypertension, hyperglycemia, obesity, and hypertriglyceridemia (McGlynn et al., 2021). Due to its much wider spread and prevalence in both adults and children globally, metabolic syndrome makes a larger contribution to the overall HCC burden than HBV or HCV infections (Sun and Karin, 2012). Patients with NAFLD-associated HCC are frequently asymptomatic. As a result, NAFLD-associated HCC tends to be more detrimental than HCC with other etiologies in many ways: elevated morbidity, more advanced stage at diagnosis, and poorer survival (Petrick et al., 2020; McGlynn et al., 2021). A 2014 meta-analysis showed that NAFLD increases the risk of HCC by 81% (Jinjuvadia et al., 2014). NAFLD encompasses a broad spectrum of liver conditions, ranging from simple hepatic steatosis, also called nonalcoholic fatty liver (NAFL), to the progressive form-nonalcoholic steatohepatitis (NASH). NASH is characterized by steatosis, inflammation, tissue damage, and reparative fibrosis. Fibrosis and cirrhosis are both known risk factors for HCC. Although extensive evidence has revealed that multiple insults, including lipotoxicity, oxidative stress, inflammation, cell death, and endoplasmic reticulum (ER) stress, all contribute to the progression from NAFLD to HCC, which is typically accompanied by cirrhosis or severe fibrosis, a growing number of NAFLD patients without advanced fibrosis or cirrhosis were found to end up developing HCC (Friedman et al., 2018; Grohmann et al., 2018). These observations suggest that there are tumor-promoting factors that act from the outset of the NAFLD. Many lines of evidence from preclinical and human studies suggest that cholesterol is independently associated with the development of cirrhosis and HCC (Matsuzawa et al., 2007; Ioannou et al., 2009; Subramanian et al., 2011; Van Rooyen et al., 2011). It is well documented that hepatic free cholesterol is a critical pathogenic factor promoting HCC through the action in multiple hepatic cell types, subcellular organelles, and molecular targets. However, a substantial body of human clinical trials found that a higher level of serum cholesterol was associated with a reduced risk of HCC. Likewise, cholesterol is closely related to the outcomes of HCC patients. Low cholesterol levels might indicate a worse disease-free and overall survival for HCC patients (Jiang et al., 2016), whereas hypercholesterolemia relates to lower future HCC mortality (Chiang et al., 2014). The contradictory observations between preclinical and population-based prospective studies implies that the relationship between cholesterol metabolism and HCC is complex and needs more careful interpretation. The exact mechanism underlying this phenomenon is still unclear. Deciphering the causal relationship between the dysregulation of cholesterol homeostasis and HCC, and elaborate on the impact of dysregulated cholesterol on specific cell types in the liver is of utmost importance to our understanding of the precise role of cholesterol in HCC development.

In view of the strong association of cholesterol metabolism with HCC and the vague understanding of the role of cholesterol in HCC pathogenesis, this review discusses the current understanding of cholesterol metabolism in HCC, providing a critical assessment of the preclinical studies, clinical trials, and the cellular and molecular regulation by cholesterol in HCC. Insights into these key elements should ultimately help understand the precise role of cholesterol and evaluate the clinical values of cholesterol in the pathogenesis of HCC.

## Cholesterol Metabolism in Physiology and Pathology

Cholesterol homeostasis is essential for health. Cholesterol serves as a precursor of various steroid hormones, bile acids, and vitamin D. Besides, cholesterol is an essential component of cell membrane and provides lipid for cell proliferation. Moreover, cholesterol controls the stability and fluidity of the cell membrane, thereby plays critical roles ranging from membrane trafficking to signal transduction (Maxfield and Tabas, 2005). Therefore, the stringent regulation of cholesterol homeostasis is vital to maintain normal physiology, and organisms have various mechanisms for accomplishing this.

**FIGURE 1:**
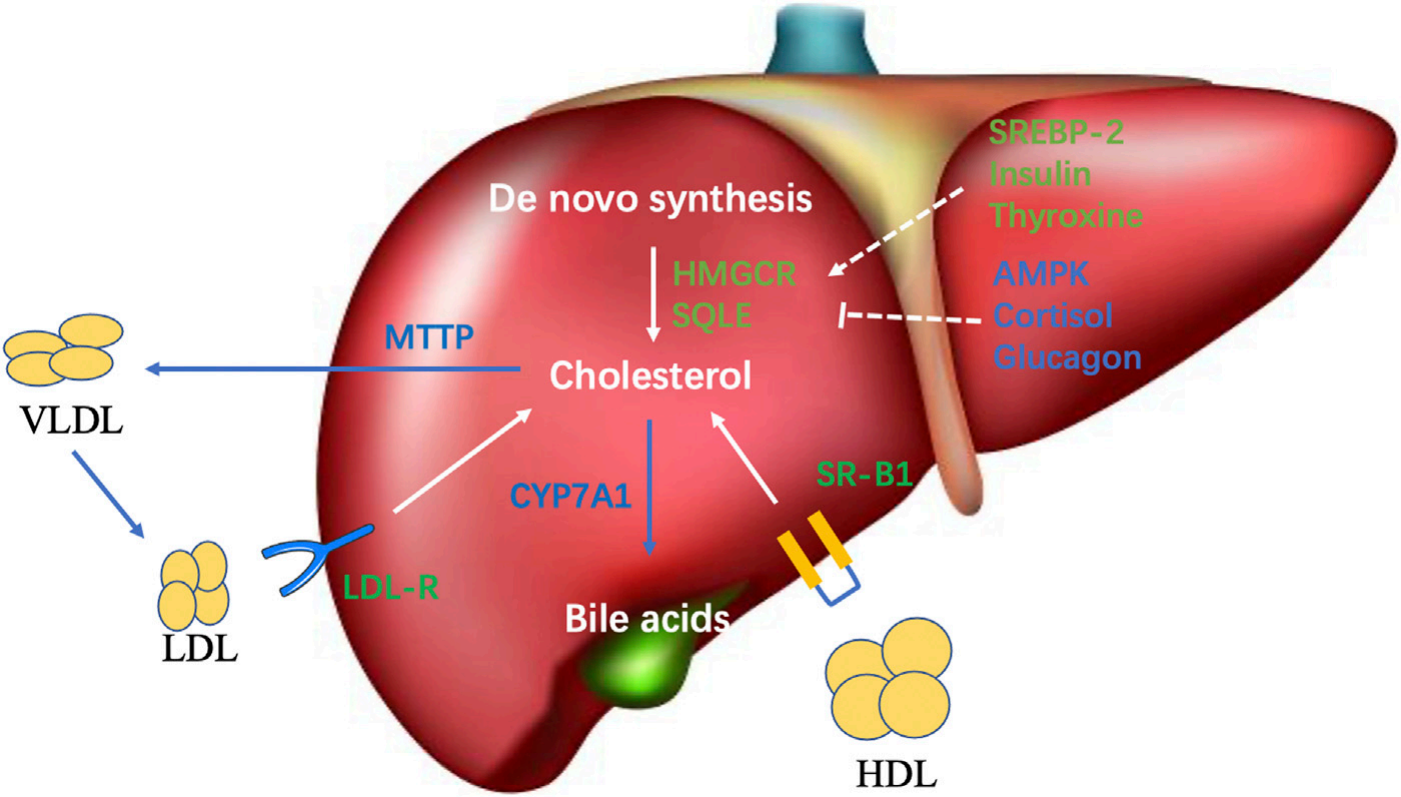
Overview of cholesterol metabolism in the liver. The primary sources of hepatic cholesterol are *de novo* synthesized and circulating cholesterol carried by apoB-containing lipoprotein particles. Output pathways of hepatic cholesterol mainly comprise VLDL secretion and bile acid synthesis.

Maintaining cholesterol homeostasis is achieved through balancing between input and output pathways, including cholesterol synthesis, dietary absorption, and excretion. The liver is the central organ in charge of cholesterol homeostasis. The primary sources of cholesterol in humans are *de novo* synthesis (∼70%) and dietary intake (∼30%) (Kapourchali et al., 2016). The liver represents the primary organ of cholesterol synthesis as it produces the majority of the *de novo* synthesized cholesterol, although the intestine also forms a significant amount (Berg JM and Stryer, 2002). Cholesterol synthesis involves complex biochemical reactions and many different enzymes, and is subjected to both transcriptional and posttranslational regulations-mediated negative feedback in response to the level of cellular cholesterol (Trapani et al., 2012). 3-hydroxy-3-methyl-glutaryl CoA reductase (HMGCR) and squalene monooxygenase (SQLE) are both rate-limiting enzymes in cholesterol biosynthesis (Figure 1). A decrease of cellular cholesterol activates the master transcription factor Sterol regulatory element-binding protein isoform 2 (SREBP-2) to induce the transcription of both HMGCR and SQLE (Pai et al., 1998; Trapani et al., 2012; Brown et al., 2018). Conversely, elevated cholesterol levels have been demonstrated to promote the ubiquitination of HMGCR and SQLE and their subsequent degradation (Gill et al., 2011; Foresti et al., 2013; Zelcer et al., 2014). AMP-activated protein kinase (AMPK) is known to phosphorylate and inactivate HMGCR, and inhibit the transcription of *SQLE*, thereby reducing cholesterol synthesis. In NASH state, the overloaded energy, insulin resistance, and inflammation synergistically inhibit AMPK, which can cause the derepression of the activity of HMGCR and the transcription of *SQLE* to enhance cholesterol synthesis (Clarke and Hardie, 1990; Sato et al., 1993; Zhang et al., 2019; Zhao et al., 2020). Insulin and thyroxine upregulate HMGCR in normal conditions. On the contrary, cortisol, and insulin counterregulatory hormones, such as glucagon, have an inhibitory effect on HMGCR (Craig and Dimri, 2020). Statins, the inhibitors of HMGCR, are widely used first-line drugs to lower plasma cholesterol and significantly decreased the risk of cardiovascular disease (CVD) and HCC in some patients. Interestingly, the CVD benefit of statins is significantly greater in patients with NASH-associated liver damage than that in patients with normal liver function. This finding is in line with the fact that CVD is the major cause of death for patients with NAFLD (Athyros et al., 2010).

Intestinal cholesterol absorption represents another important cholesterol input source to maintain cholesterol homeostasis. Cholesterol uptake by small-intestinal enterocytes is mainly mediated by the specific transporter protein Niemann-Pick C1 Like 1 (NPC1L1), which mainly locates on the brush-border membrane of enterocytes. Cholesterol is esterified by acetyl-CoA cholesterol acyltransferase 2 (ACAT-2) and assembled into chylomicrons together with triglycerides and apolipoprotein B-48. Triglycerides carried by chylomicrons are then absorbed by peripheral tissues. The chylomicron remnants are taken up by the liver. Ezetimibe, which inhibits intestinal cholesterol absorption, was found to improve NASH in human trials (Yoneda et al., 2010; Park et al., 2011).

Besides *de novo* synthesis of cholesterol, the liver also uptakes excess circulating cholesterol carried by lipoprotein particles, including low-density lipoprotein (LDL), intermediate-density lipoprotein (IDL), and chylomicron remnants via the LDL receptor (LDLR); and high-density lipoprotein (HDL) via the scavenger receptor class B type 1 (SR-B1) for further clearance (Maxfield and Tabas, 2005) (Figure 1). LDLR-mediated lipoprotein uptake is a major route through which the human body clears excessive blood cholesterol, whereas it is also a major cholesterol input pathway for the liver. The uptake of LDL is tightly regulated by feedback inhibition, in which increased cellular cholesterol inhibits SREBP2 to reduce the transcription of LDLR, thereby preventing cholesterol overload in the cells. However, in pathogenic conditions, the oxidized LDL (OxLDL) formed under oxidative stress can be taken up by scavenger receptors in an unregulated fashion (Levitan et al., 2010). As a result, the accumulation of OxLDL in the liver causes lipotoxicity. Moreover, the uptake of OxLDL by Kupffer cells causes increased hepatic inflammation to promote NASH and HCC (Walenbergh et al., 2013).

Free cholesterol transports between compartments of the cell, such as the plasma membrane and endoplasmic reticulum (ER). Free cholesterol can be reesterified in the ER and stored in the cytoplasm as lipid droplets. To prevent the accumulation of cholesterol in the liver, hepatocytes must clear excess cholesterol (Figure 1). One of the main pathways is through being packaged along with triglycerides and apolipoprotein B-100 into the very low-density lipoproteins (VLDL) and secreted into the circulation. VLDL delivers triglycerides and cholesterol (later forms LDL after losing triglycerides) into the peripheral tissue (Ikonen, 2008). Defect or inhibition of the microsomal triglyceride transfer protein (MTTP), the key protein for VLDL assembly, will cause lipid accumulation in the liver and promote NASH and HCC development (Ipsen et al., 2018). Another main process to export liver cholesterol is the conversion of cholesterol into bile acids by a complex enzymatic process and consequently eliminating some cholesterol. Cytochrome P4507A1 (CYP7A1) is the rate-limiting enzyme (Figure 1). Ileum enterocytes generate fibroblast growth factor 19 in humans and 15 in mice to inhibit the expression of CYP7A1 upon uptaking of bile acids.

While the liver plays a central role in whole-body cholesterol homeostasis, it is important to keep in mind that maintaining cholesterol balance in the local liver environment is equally critical to prevent liver diseases. Unfortunately, cholesterol does not always reach its most appropriate balance at both whole-body and hepatic levels. One example is the regulation of MTTP, a key protein regulating VLDL assembly and thereby transporting cholesterol from the liver into the circulation. MTTP was once the favorite target to lower plasma cholesterol, and several antagonists (Bay-13-9952, CP-346086, BMS-201308, AEGR-733) have been identified to treat atherosclerosis. However, although these early studies showed that, while MTTP inhibition effectively lowered plasma cholesterol, it significantly increased hepatic lipid and liver damage, which elevated the risk of NAFLD and HCC (Chang et al., 2002; Cuchel et al., 2007; Samaha et al., 2008).

## Cholesterol and the Pathogenesis of HCC

### Hypercholesterolemia Promote Tumorigenesis in HCC

Obesity and insulin resistance have been established as risk factors for benign NAFL; however, the cause of progressive NASH remains unclear. A recent report showed that cholesterol supplement is critical to the development of inflammation and fibrosis in mice fed with a high-fat diet (Ioannou et al., 2009). Cholesterol is an essential component of both the Amylin liver NASH (AMLN) diet and its later replacement Gubra Amylin NASH (GAN) diet to elicit NASH and fibrosis (Boland et al., 2019). Both diets have been extensively used in mouse studies to mimic human NASH. The role of dietary cholesterol in NASH was also observed in other species, such as Ossabaw pigs. The supplement of hypercholesterolemia resembled human NASH hallmarks, including steatosis, inflammation, hepatocyte damage, and fibrosis (Lee et al., 2009).

**FIGURE 2:**
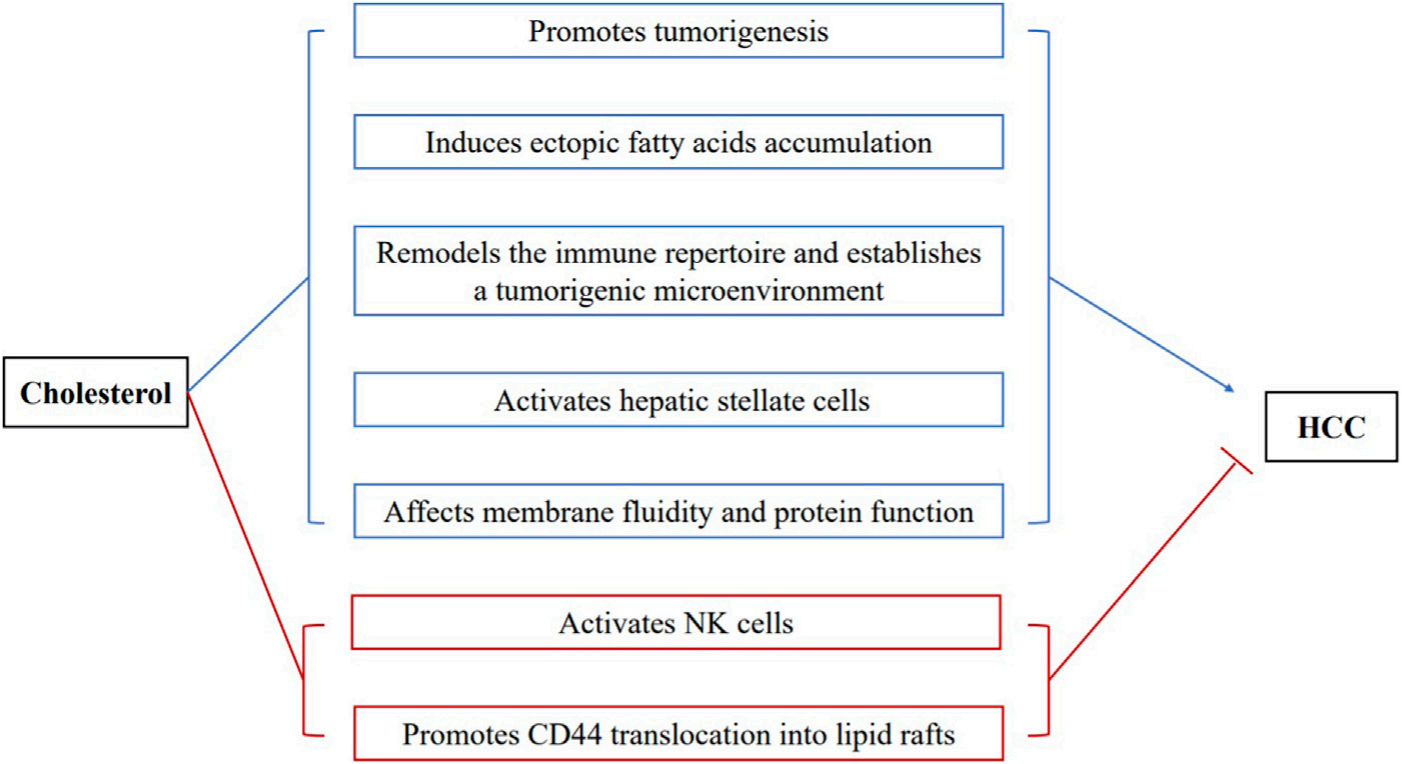
The complex roles of cholesterol in the development of HCC. Proposed mechanisms for the pathogenic roles of cholesterol in HCC are revealed (blue characters): (1) promoting tumorigenesis; (2) inducing ectopic fatty acids accumulation; (3) remodeling the hepatic immune repertoire and establishing a tumorigenic microenvironment; (4) activating hepatic stellate cells; (5) affecting membrane fluidity and protein function. Mechanisms by which cholesterol inhibits HCC development (red characters): (1) activating NK cells to fight against hepatoma cells; (2) promoting the translocation of CD44 into lipid rafts and attenuating CD44-mediated migration and metastasis of HCC.

In line with the pathogenic role of dietary cholesterol, *de novo* cholesterol synthesis also exhibits pathogenic and prognostic significance for HCC. SQLE was the top metabolic gene enriched in the liver of NAFLD associated-HCC patients (Liu et al., 2018). Overexpression of SQLE promoted the proliferation and migration of HCC cells (Sui et al., 2015). Terbinafine, an antifungal drug targeting SQLE, reduced cholesteryl ester levels and suppressed tumor growth (Liu et al., 2018). Interestingly, the downregulation of LDLR that mediates the endocytosis of LDL cholesterol, increases the risk of human HCC. The mechanistic study demonstrated that the reduced LDLR expression promoted intracellular *de novo* cholesterol biosynthesis to compensate the decreased LDL uptake (Chen et al., 2021). Altogether, these preclinical studies provide compelling evidence that cholesterol is an independent risk factor for NASH and fibrosis, and substantially increase the risk of HCC (Figure 2).

### Cholesterol Induces Ectopic Fatty Acids Accumulation, Forming a Vicious Cycle Causing Lipotoxicity, Inflammation, and Cell Injury in Hepatocytes

Hepatic lipotoxicity refers to the ectopic accumulation of triglycerides and their intermediates in the liver, which causes lipotoxic hepatocellular injury (Neuschwander-Tetri, 2010). In patients with metabolic syndrome, insulin resistance causes dysregulated lipolysis of adipose tissue and increased delivery of free fatty acids to the liver. In hepatocytes, excessive fatty acids lead to energy overload, together with insulin resistance and inflammation, which are frequently seen in metabolic syndrome, causes repression of the energy sensor, AMPK (Zhao et al., 2018). A recent study showed that repressed AMPK lost its inhibitory capability on caspase 6 in normal livers. Consequently, the sustained activation of caspase 6 induced cell death and eventually liver fibrosis (Zhao et al., 2020). Suppressed AMPK also releases its inhibition on HMGCR and enhances cholesterol synthesis in the hepatocytes. It has been shown that cholesterol crystals are formed due to the hydrolysis of excess cholesterol easter into free cholesterol by lysosomal acid lipase (Ioannou, 2016). These cholesterol crystals can activate the NLRP3 inflammasome and subsequent inflammation in hepatocytes. The activated NLRP3 inflammasome further results in pyroptosis by inducing caspase 1 activation and Gasdermin-mediated pore formation in the cell membrane. At the tissue level, subsequent tissue repair and remodeling occur and lead to fibrosis and HCC (Bergsbaken et al., 2009; Miao et al., 2011; Dixon et al., 2012; Wree et al., 2014; Szabo and Iracheta-Vellve, 2015). Dietary cholesterol has been shown to downregulate hepatic cholesterol ester and lipoprotein synthesis, therefore, in turn, suppresses hepatic triglyceride secretion to promote NASH and HCC (Ma et al., 2014; Henkel et al., 2017). Hypercholesterolemia is often accompanied by the elevation of OxLDL. The increased uptake of OxLDL by hepatocytes can lead to the accumulation of oxidized phospholipids (OxPLs), key pathogenic component of OxLDL. Recently, OxPLs have been proved to cause NASH and HCC through decreased mitochondrial fatty acid oxidation, thereby lead to fatty acid accumulation and lipotoxicity (Sun et al., 2020). Conclusively, cholesterol forms a feed-forward vicious cycle with ectopic fatty acid accumulation to cause tumorigenic detrimental effects, including cell damage, inflammation, and fibrosis (Figure 2).

### Cholesterol Remodels the Immune Repertoire in the Liver and Establishes a Tumorigenic Microenvironment

Inflammation is a crucial component of tumor progression (Coussens and Werb, 2002). Similar to that in hepatocytes, cholesterol crystal induces a more robust NLRP3 inflammasome activation in Kupffer cells. Moreover, the resident Kupffer cells became foam cells upon taking up OxLDL and OxLDL-containing dead hepatocytes. Together with activated hepatocytes, these Kupffer cells produce a panel of inflammatory cytokines/chemokines to recruit a variety of inflammatory immune cells. In particular, recruited macrophages facilitate a tumorigenic cascade in the liver microenvironment by producing a large amount of cytokines, chemokines, growth factors, and triggering the release of inhibitory immune checkpoint proteins by T cells, which are all critical features of tumor-associated macrophages (Lin et al., 2019). As a result, the recruited immune cells replenished the niche of the dead foam cells and hepatocytes and established an inflammatory tumorigenic microenvironment (Seidman et al., 2020) (Figure 2).

### Cholesterol Induces the Activation of Hepatic Stellate Cells

Hepatic stellate cells (HSCs) are the key cell type responsible for liver fibrogenesis. Upon activation, HSCs differentiate into myofibroblasts and produce fibrogenic growth factors and secrete extracellular matrix to promote liver fibrosis (Trivedi et al., 2021). Cholesterol activates HSCs through multiple mechanisms. Cholesterol crystal-activated Kupffer cells and OxLDL-loaded foam cells could elicit HSC activation through inflammatory cytokines (Kolios et al., 2006). Recent evidence suggests that free cholesterol can directly activates HSCs (Tomita et al., 2014). Moreover, OxLDL can activate HSCs through TLR4 -dependent pathway. Most recently, it is shown that OxPLs can directly activate the fibrogenic gene expression in HSCs (Sun et al., 2020). The activated HSCs are then differentiated into myofibroblasts and accelerate fibrosis (Figure 2).

### Cholesterol Content Affects Membrane Fluidity and Protein Function

Cholesterol plays a critical role in maintaining membrane fluidity. An increase of membrane cholesterol concentration in metabolic syndrome may, in turn, disrupt the normal function of the plasma membrane, and membrane of different organelles. Lipid raft proteins, such as Toll-like receptor 4 (TLR4), have been shown to be activated to enhance the inflammatory response in cholesterol-rich membranes (Zhu et al., 2010). Increased mitochondrial cholesterol reduces mitochondrial membrane fluidity and impairs the electron transport chain (ETC), and lead to increased ROS generation, lipid peroxidation, hepatocyte necrosis, and apoptosis, which are all known HCC risk factors (Martin et al., 2016; Solsona-Vilarrasa et al., 2019). An increase of ER membrane cholesterol is known to result in ER stress (Tabas, 2010). Cholesterol overload on lipid droplet membrane can induce cholesterol crystallization, thereby induce the activation of NLRP3 inflammasome (Ioannou et al., 2017; Ioannou et al., 2019). Collectively, cholesterol loading on the membrane system can increase the risk of HCC through a multi-level mechanism (Figure 2).

### Cholesterol-Lowering Drugs and HCC

Table 1 shows the new approaches that target cholesterol metabolism in HCC. The cholesterol-lowering drug Ezetimibe was shown to reduce the serum aminotransferases levels, hepatic steatosis, and hepatocyte ballooning in NASH (Nakade et al., 2017). Many studies demonstrated that statins, the HMGCR inhibitors, might protect against the development and recurrence of HCC (Friedman et al., 2016; Kim et al., 2018; German et al., 2020; Tran et al., 2020). In addition to their cholesterol-lowering effects, statins exhibit multiple pleiotropic effects on the development of HCC, including anti-inflammatory, antifibrotic, antiproliferative, and endothelial protection effects (Kim and Kang, 2019). However, not all studies came to the same conclusion. In a mean prospective follow-up of 8.4 years study, statin users had a 40% lower risk of HCC in a total cholesterol-unadjusted analysis. The association disappeared after adjusting the influence of cholesterol level measured within 6 months before statin initiation (Yi et al., 2020). This study showed that high cholesterol levels at statin initiation were associated with the high risk of HCC. A recent meta-analysis, which includes twenty-five studies with 1,925,964 patients, showed that statin reduces the risk of HCC by 26%. This effect is dose-dependent and more pronounced with lipophilic statins (atorvastatin, lovastatin, and simvastatin) (Facciorusso et al., 2020).

**TABLE 1:** New approaches that target cholesterol metabolism in HCC.

Drugs	Target	Clinical development	Effects	References
Statins	HMGCR	In phase II clinical trials	Controversial	70–79
Ezetimibe	NPC1L1	In preclinical development	Suppressed development of liver tumor	69, 80
Terbinafine	SQLE	In preclinical development	Attenuated HCC in cell and mice model	43, 46

The inconclusive results suggest that cholesterol lowering drugs might have distinct roles for HCC with different etiology. Recent studies revealed that in viral hepatitis-associated HCC, statins increase the response rate to antiviral therapy, reduce the incidence of liver fibrosis, and prevent the occurrence of HCC in HBV and HCV patients (Butt et al., 2015; Hsiang et al., 2015). However, in patients with NAFLD-associated HCC, it was found that statins did not reduce the overall incidence of HCC (Yi et al., 2020). In a preclinical study, atorvastatin failed to reduce the incidence of HCC in mice exposed to N-nitrosodiethylamine (Braeuning et al., 2014). Ezetimibe was proven to suppress the development of liver tumors by inhibiting angiogenesis in *Pten* ^Δhep^ mice with hypercholesterolemia (Miura et al., 2019). *SQLE* is the top gene correlated with NAFLD-associated HCC in patients. Terbinafine, an inhibitor of SQLE, markedly inhibited HCC cell growth in xenograft models in *Sqle* transgenic mice (Liu et al., 2018). Collectively, these data indicate that lowering cholesterol has the potential to ameliorate HCC in patients with certain etiology. The contradictory results from statin treated patients and terbinafine treated mice are likely affected by multiple factors, including the timing of intervention and the target of different drug. For instance, statins mainly work through inhibition of cholesterol synthesis to upregulate SREBP2 activity to ultimately increase LDLR to promote LDL cholesterol clearance by the liver. In this case, statins, in fact, increase cholesterol load into the liver, which could partially explain its lack of anti-HCC effect.

### Roles of Different Types of Cholesterol in HCC

As cholesterol-lowering plays a central role in atherosclerotic cardiovascular disease therapy, it could be of tremendous value to understanding the role of cholesterol in HCC of certain patients with high cardiovascular risk. Both HCC and cardiovascular diseases are life-threatening co-morbidities of NAFLD (Brunt et al., 2015), and closely related to cholesterol metabolism. However, the relationship between cholesterol and cardiovascular disease as well as HCC is in fact more complicated due to the dominant roles of distinct subtypes of cholesterol in different conditions. The consensus among the world is that LDL cholesterol is a critical pathogenic factor and therapeutic target of cardiovascular diseases (Ference et al., 2017). Meanwhile, cardiovascular disease is twice as likely the cause of death of patients with NAFLD than other liver diseases. This seems likely related to commonly shared risk factors including high LDL cholesterol. However, a recent clinical study found that HDL cholesterol but not LDL cholesterol was significantly associated with the HCC Tumor Aggressiveness Index. HDL cholesterol had a statistically higher hazard ratio for death than LDL cholesterol in HCC patients (Carr et al., 2018). On the contrary, an earlier study found that an increased HDL cholesterol level was related to improved overall survival (HR, 0.679, *p* < 0.01) and disease-free survival (HR, 2.085, *p* = 0.002) rate (Jiang et al., 2016). One possible explanation is that LDL and HDL play different roles during cholesterol transportation. In the context of cardiovascular disease, elevated LDL cholesterol in the circulation penetrates the vascular wall and being taken up by macrophages to form foam cells, which is the hallmark of atherosclerotic plaques. On the contrary, HDL-C removes redundant cholesterol from the vascular cells to maintain normal cell cholesterol homeostasis and prevent atherosclerosis (Glass and Witztum, 2001). In HCC conditions, since HDL serves as the reverse cholesterol transporter that carries peripheral cholesterol back to the liver through HDL receptor-scavenger receptor B type I (SR-BI), it could thus promote tumorigenesis. With this regard, another role of HDL is carrying the inflammatory oxidized phospholipids to the liver through SR-BI, which could also promote HCC. The lack of association between LDL cholesterol with HCC might also be affected by the patient’s other condition, such as their LDLR level. LDL particles carry cholesterol to all cells with LDLR. Although the liver is the primary source of cholesterol clearance, the LDLR is frequently downregulated in hepatocytes in some NAFLD patients. The observation from the earlier study that HDL related to improved survival rate can also be due to patients’ cardiovascular condition. Given that cardiovascular diseases exhibit a leading cause of death in NAFLD patients compared to HCC, higher HDL levels could improve overall survival rate by improving cardiovascular conditions. Therefore, to conclude an explicit role of cholesterol with HCC, the kind of cholesterol as well as other conditions, such as cardiovascular diseases, need to be characterized in the patients.

## Clinical Observation

The relationship between total cholesterol and the risk of liver cancer in human trials is controversial. A substantial body of work found that a higher level of serum cholesterol is associated with a lower risk of HCC (Ahn et al., 2009; Iso et al., 2009; Kitahara et al., 2011; Yi et al., 2020; Cho et al., 2021). A large prospective study, including 1,189,719 adults, showed that elevated total cholesterol was associated with a lower incidence of liver cancer (Kitahara et al., 2011). The results of liver cancer is consist of two other prospective cohort studies performed in Japan and Korea. The Japanese study included 33,368 men and women aged 40–69 years, and the average follow-up period was 12.4 years (Iso et al., 2009). Serum total cholesterol levels were inversely related to the risk of liver cancer in both sexes, and the inverse association remained after exclusion for the first 3-years incident cases and advanced cases with metastasis (Iso et al., 2009). The other study followed 400,318 Koreans for an average of 8.4 years, and there were 1,686 individuals diagnosed with HCC. The result demonstrated that high cholesterol levels were associated with a lower risk of HCC (Yi et al., 2020). In contrast, the inverse association between total serum cholesterol was no longer significant in a cohort of Finnish male smokers after excluding the cases diagnosed during the first 9 years of follow-up (Ahn et al., 2009). Cholesterol is also related to the outcomes of HCC patients. Low cholesterol levels might predict worse disease-free survival and overall survival for HCC patients (Jiang et al., 2016). Hypercholesterolemia was inversely related to HCC mortality (Chiang et al., 2014).

The results of these prospective studies imply that the relationship between cholesterol metabolism and HCC is far more complex in humans. The exact mechanism is still unclear, and we made several hypotheses to explain the adverse association between cholesterol level and HCC that is opposite to preclinical studies. First, similar to albumin, aminotransferases, and other protein that hepatocytes produce, low cholesterol levels may reflect impaired hepatic function as impaired hepatocytes could no longer maintain their cholesterol-generating capability. This is supported by the fact that cholesterol is negatively associated with the severity of liver cirrhosis, in which normal hepatocyte functions are severely impaired (Krautbauer et al., 2017). These chronic hepatic diseases may exaggerate the reverse relationship between hypercholesteremia and HCC incidence. Second, the serum cholesterol was uptaken by hepatoma cells to meet the high cholesterol demand of cancer cells. In microsomes isolated from hepatomas, the level of cholesterol was about 30% higher than the value of normal hepatic cells surrounding the tumors (Eggens et al., 1990; Su et al., 2004). Therefore, lower blood cholesterol is observed in patients with more active HCC. Third, hypercholesterolemia per se suppresses the incidence and development of HCC. Though most preclinical studies support the pathogenic role of cholesterol in HCC development, some studies imply that high levels of cholesterol protect against HCC. There is a growing body of evidence supporting that cholesterol plays a vital role in regulating immune cell function (Kopecka et al., 2020). Recently studies showed that cholesterol accumulation in NK cells could activate the effector functions of NK cells against hepatoma cells (Qin et al., 2020). Lipid rafts, cholesterol-enriched membrane domains, play a critical role in the regulation of signaling transduction in cancers (Pike, 2009; Greenlee et al., 2021). High levels of cholesterol promoted CD44 translocation into lipid rafts and attenuated CD44-mediated migration and metastasis of HCC (Yang et al., 2018) (Figure 2).

## Discussion

Cholesterol homeostasis plays a vital role in the normal functions of organs, cells, and proteins. The precise role of cholesterol in the development of HCC is complex and needs to be addressed according to disease stage, cell type, HCC etiology, types of cholesterol, concurrent diseases, and many other factors. Extensive preclinical studies have proved a tumorigenic role of hepatic cholesterol in promoting the transition from NASH to HCC. Cholesterol acts as a risk factor through a multi-level mechanism, ranging from tissue microenvironment, cellular, and molecular regulations. However, in the advanced stage of HCC, when extensive liver damage is established, a higher cholesterol might indicate a better-preserved liver function. In this scenario, studies might conclude an opposite association between cholesterol and HCC. Moreover, in established HCC, increased intracellular cholesterol might play a detrimental role in one cell type, tumor cell, for instance, but may promote the immune surveillance function of immune cells, thereby exhibit overall beneficial effects. The etiology of HCC might also affect the association between cholesterol and HCC outcome. Whether HCC was caused by hepatitis virus infection, NAFLD, alcoholic liver diseases, or other conditions needs to be considered. Given the controversial observation of the roles of subtypes of cholesterol in HCC outcome, a careful evaluation on patients’ cholesterol profile could offer more accurate evidence on the roles of cholesterol in HCC. Another factor that needs to be considered is the cholesterol levels in circulation versus that in the liver. The inconsistency between blood and liver cholesterol might affect the conclusion. This was supported by the contradictory effect of statins in HCC progression. Owing to the central role of liver in cholesterol homeostasis, cholesterol lowing drugs that increase cholesterol loading into the liver might need more careful evaluation when applied to HCC therapy. HCC patients often have concurrent other disease conditions, such as cardiovascular diseases, which are ranked top cause of death for patients with NAFLD compared to HCC. Therefore, for patients with cardiovascular diseases, subtypes of cholesterol might predict a different outcome depending on their roles in cardiovascular conditions. Altogether, a rigorous assessment of all these factors might help future clinical trial designs. When assessing the roles of cholesterol in HCC development or HCC therapy, the cholesterol lowering drugs need to be carefully compared owing to their action target.

## Conclusion

Cholesterol homeostasis is essential to health. Hypercholesterolemia is getting more and more attention from researchers as it exhibits associations with HCC. To draw a definitive conclusion of the relationship between cholesterol and HCC, many associated factors need to be taken into consideration for future clinical trial design, including etiology of HCC, detailed cholesterol profile including HDL and LDL, HCC stage, cholesterol-lowering drugs that are being used and their mechanisms of action, and their other conditions such as cardiovascular disease. Given the central role of cholesterol in atherosclerotic cardiovascular disease, the increasing health threat that HCC has brought to global populations, clinical studies that determine the role of cholesterol in HCC have great potential to shed some lights on our current understanding of HCC pathogenesis and therapy.
